# Supplementary material for: Damage Effect of Amorphous Carbon Black Nanoparticle Aggregates on Model Phospholipid Membranes: Surface Charge, Exposure Concentration and Time Dependence
Source: Int J Environ Res Public Health. 2023 Feb 8;20(4):2999. doi: 10.3390/ijerph20042999 (PMC9959192; doi:10.3390/ijerph20042999)
Supplement: Supplementary file 1 [file ijerph-20-02999-s001.zip › ijerph-2152936-supplementary.pdf]

Supporting information

Damage Effect of Amorphous Carbon Black Nanoparticle Aggregates on Model Phospholipid Membranes: Surface Charge, Exposure Concentration and Time Dependence

Xiao-feng Wang, Kun Xu, Xin-rui Li, Ya-xin Liu and Jie-min Cheng *

College of Geography and Environment, Shandong Normal University, Jinan 250358, China

* Correspondence: jmcheng2002@hotmail.com.; Tel.: +86-0531-86182550; Fax: +86-0531-86180604

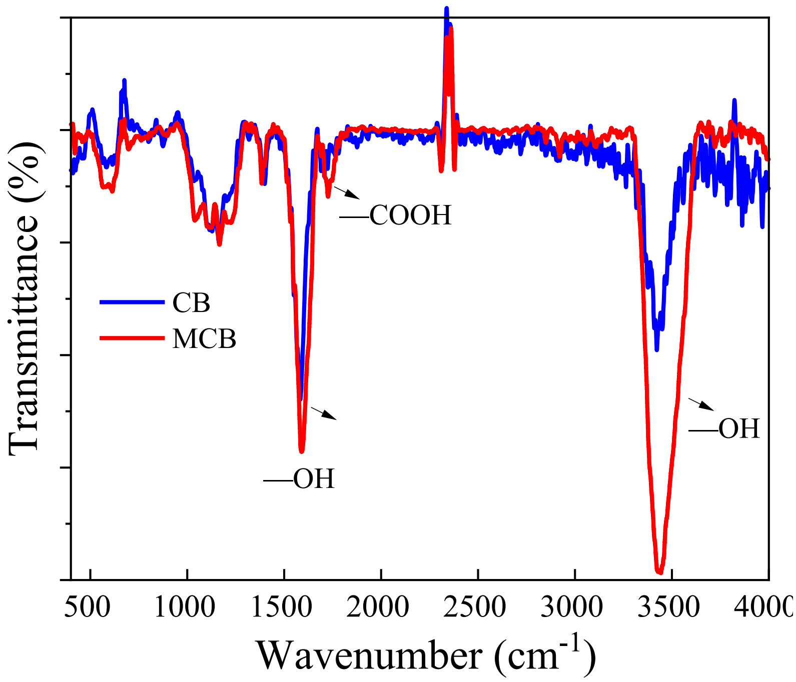

Figure S1. FTIR spectra of CB and MCB.
